# A symmetric systemic challenge elicits a right‐biased response mediated by vasopressin signaling

**DOI:** 10.1111/jne.70257

**Published:** 2026-09-06

**Authors:** Hiroyuki Watanabe, Yaromir Kobikov, Sara Yusuf Mohamed, Karen Rich, Daniil Sarkisyan, Olga Nosova, Alfhild Grönbladh, Mathias Hallberg, Jens Schouenborg, Georgy Bakalkin, Mengliang Zhang

**Affiliations:** ^1^ Department of Pharmaceutical Biosciences Uppsala University Uppsala Sweden; ^2^ Department of Molecular Medicine University of Southern Denmark Odense M Denmark; ^3^ Department of Immunology, Genetics and Pathology and Science for Life Laboratory Uppsala University Uppsala Sweden; ^4^ Neuronano Research Center, Department of Experimental Medical Science Lund University Lund Sweden

**Keywords:** functional asymmetry, left–right balance, symmetric systemic challenge, vasopressin, water deprivation

## Abstract

Bilaterian animals exhibit functional asymmetry—population‐level, directional left–right differences in physiology and behavior, including responses to spatially symmetric environmental challenges. Whether such symmetry‐to‐asymmetry conversion can be driven at the systems level by neurohormonal regulators remains unclear. Here we tested whether a spatially symmetric neuroendocrine challenge—water deprivation (WD)—can elicit a directional left–right physiological response in rats using hindlimb postural asymmetry (HL‐PA), a binary readout that quantifies left‐ versus right‐sided hindlimb flexion. Twenty‐four hours of WD induced robust HL‐PA with right hindlimb flexion, revealed under anesthesia. The asymmetry persisted after complete thoracic spinal cord transection, suggesting that humoral signaling, rather than descending neural commands, maintains the postural bias. Dehydration activated the hypothalamic arginine vasopressin (AVP) system. Furthermore, a V1B antagonist (SSR‐149415) and a V1A/V2 antagonist (conivaptan) abolished and partially attenuated WD‐induced HL‐PA, respectively, supporting an AVP‐dependent mechanism that likely operates at least two anatomical sites. AVP signaling may involve pituitary V1B‐dependent endocrine output and spinal V1A actions; consistent with the latter, expression of AVP V1A receptors is right‐biased in lumbar spinal cord. Together, these findings identify WD as a symmetric systemic challenge capable of unmasking a directional peripheral bias and implicate vasopressin signaling in left–right physiological regulation. More broadly, they suggest that symmetric homeostatic challenges engage neuroendocrine mechanisms that shift the balance between left‐ and right‐sided physiological functions.

## Graphical abstract

Water deprivation, a spatially symmetric homeostatic challenge, induces vasopressin receptor‐dependent right‐sided hindlimb asymmetry in rats, providing a binary readout of lateralized physiological regulation. These findings suggest that symmetric homeostatic challenges can engage neuroendocrine mechanisms that shift the balance between left‐ and right‐sided physiological functions.

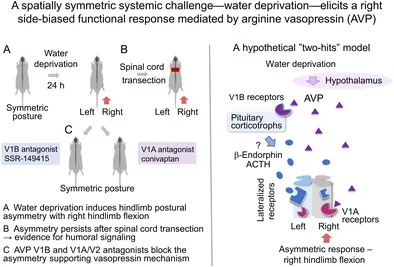

## 1 INTRODUCTION

Alongside developmental left–right asymmetry (e.g., visceral laterality established during embryogenesis),^1^, ^2^ bilaterian animals also exhibit functional asymmetry—population‐level, directional differences in physiological processes between the generally symmetric left and right regions of nervous system.^3^, ^4^ This functional asymmetry may enable bilaterian systems to transform symmetric external inputs into asymmetric outputs through lateralized internal processing, following the general scheme “symmetric stimulus → symmetric gross anatomy → asymmetric response.”^3^, ^4^, ^5^ For example, in *Caenorhabditis elegans*, salt changes are encoded asymmetrically by paired gustatory neurons, enabling directional chemotaxis in a symmetric stimulus field.^5^

It remains unclear whether such left–right‐biased processing is restricted to particular sensory cues or instead reflects a broader principle of nervous system organization that has been insufficiently explored, especially in vertebrates. It is also unknown whether these asymmetric responses arise only from local anatomical specialization or can be implemented at the systems level by systemic regulators, particularly neurohormones and neuropeptides.^6^ These signaling molecules have recently emerged as local and systemic regulators of lateralized brain functions and of the balance between mirror‐symmetric neural systems.^6^, ^7^, ^8^, ^9^, ^10^, ^11^, ^12^, ^13^, ^14^, ^15^, ^16^, ^17^, ^18^, ^19^ In *C. elegans*, peptide hormone signaling reshapes laterality under symmetric conditions: INS‐6 insulin signaling remodels salt‐circuit computations.^7^ In rodents, neuropeptides and neurohormones can also modulate and induce side‐specific responses.^6^ For example, signaling by dynorphin, calcitonin gene‐related peptide, and substance P is lateralized in the parabrachial‐to‐amygdala circuit that underlies asymmetric pain processing.^12^, ^13^, ^14^, ^15^, ^16^ Brain injury studies further reveal side‐specific functions of arginine vasopressin (AVP) and opioid peptides as endocrine mediators of contralateral effects on posture and motor function.^20^, ^21^, ^22^, ^23^, ^24^, ^25^ Lesions of the left sensorimotor cortex produce hindlimb postural asymmetry (HL‐PA) with right hindlimb flexion, an effect blocked by the AVP antagonist SSR‐149415, whereas intravenous AVP mimics this contralateral response in animals with intact brains.

Here, we asked whether a symmetric systemic neuroendocrine challenge can generate a population‐level directional left–right physiological output, and whether a neurohormone with known side‐specific actions, such as AVP, can mediate this transition from symmetry to asymmetry. We therefore tested whether water deprivation (WD) can elicit HL‐PA as a directional left–right response and whether this response depends on AVP signaling. WD lacks inherent directional cues, such that any left–right bias would argue for internally generated neuroendocrine symmetry breaking, and it robustly recruits the hypothalamic–neurohypophysial AVP system.^26^, ^27^, ^28^ As a readout for lateralized endocrine responses, we employed the HL‐PA model—a binary outcome system that quantifies left‐ versus right‐sided postural changes induced by neurohormones.^21^, ^22^ To isolate neuroendocrine signaling from descending neural input, the spinal cords were completely transected at the thoracic level in a subset of the experiments.

## 2 MATERIALS AND METHODS

### 2.1 Animals

Wild‐type male Wistar rats were obtained from Janvier labs, France. The animals received food and water ad libitum and were maintained in a 12‐h day‐night cycle (light on from 10:00 p.m. to 10:00 a.m.) at a constant environmental temperature of 21°C (humidity: 65%). Ethical approval was obtained from the Malmö/Lund ethical committee of animal experiments (Dnr. 5.8.18‐17317/2021). Rats with pre‐experimental body weights ranging from 147 to 474 g were used. The sample sizes for each experimental group are indicated in the corresponding figure legends and in Table S1, Supporting Information. This table also summarizes body‐weight ranges for the individual experimental groups. Exact age records were not available for all animals.

### 2.2 Experimental design and drug treatment

**FIGURE 1:**
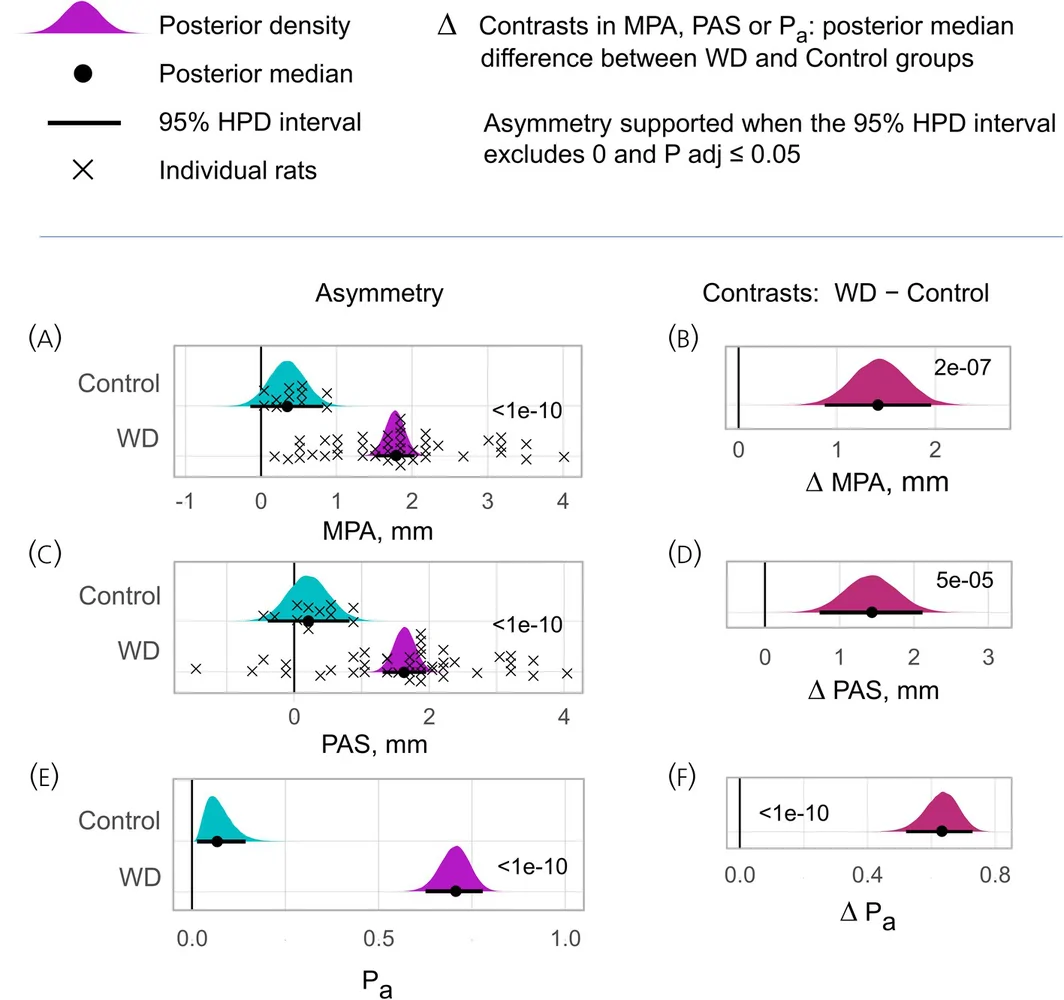
Water deprivation (WD)–induced hindlimb postural asymmetry (HL‐PA). Magnitude of postural asymmetry (MPA), postural asymmetry score (PAS), and the proportion of asymmetric animals (P_a_) were quantified. PAS encodes directionality: negative values indicate left hindlimb flexion and positive values indicate right hindlimb flexion. (A, C, E) HL‐PA in control (*n* = 11) and water‐deprived (*n* = 43) rats (Table S1); crosses denote individual animals. (B, D, F) Between‐group contrasts (water‐deprived minus control) for MPA (ΔMPA), PAS (ΔPAS), and P_a_ (ΔP_a_). Posterior summaries from Bayesian regression are shown as posterior medians (black circles), 95% highest posterior density (HPD) intervals (black horizontal lines), and posterior distributions (colored density plots). Effects were considered supported when the 95% HPD interval excluded zero (MPA ≠ 0, PAS ≠ 0 and P_a_ ≠ 0 in A, C, E; ΔMPA ≠ 0, ΔPAS ≠ 0 and ΔP_a_ ≠ 0 in B, D, F) and the corresponding multiplicity‐adjusted *p*‐value was ≤.05 (adjusted *p*‐values are shown on the plots). Credible intervals are the primary inferential summaries; adjusted *p*‐values are reported as supplementary model‐based estimates.

**FIGURE 2:**
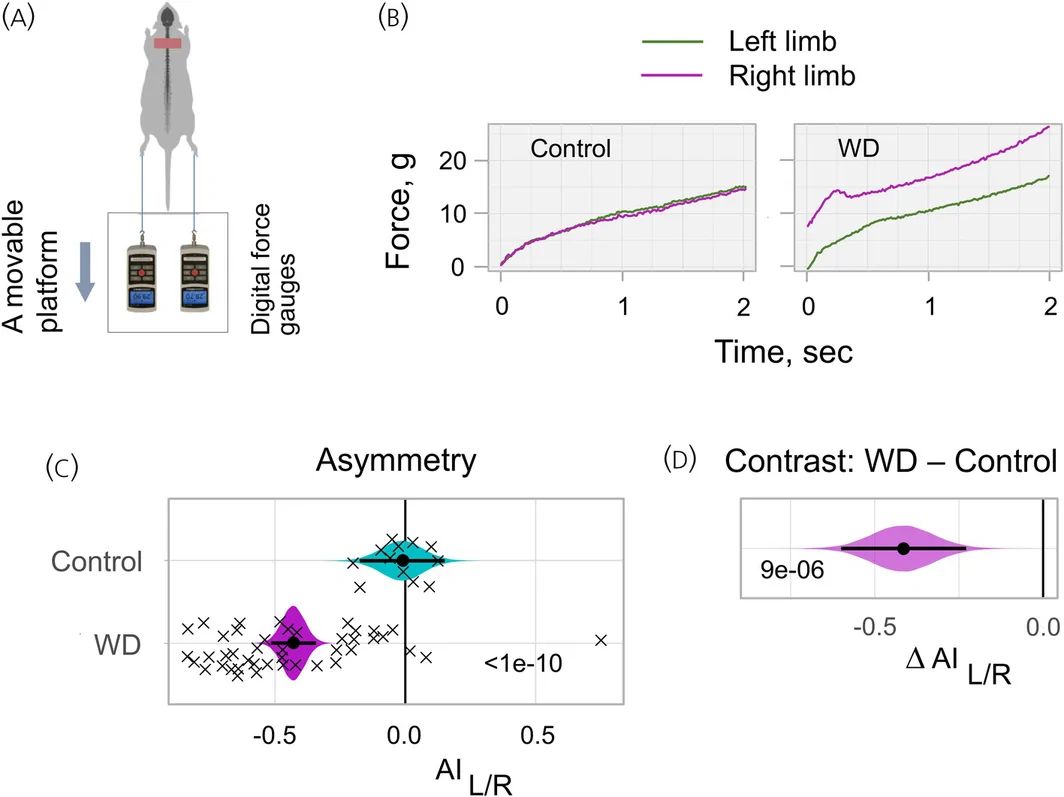
Effects of WD on hindlimb stretching resistance. Hindlimb stretching resistance was quantified as the mechanical work (W, g × mm) required to stretch a hindlimb over 0–10 mm within 2 s. Asymmetry was expressed as AI_L/R_ = log_2_(W_L_/W_R_), where W_L_ and W_R_ are the work values for the left and right hindlimbs, respectively. Group designations and treatment protocols are described in Figure 1. (A) Schematic of the custom micromanipulator‐based device used to record hindlimb stretching force, incorporating dual digital force gauges mounted on a movable platform. (B) Representative force traces recorded from the left and right hindlimbs of control and WD rats. (C) AI_L/R_ values in control (*n* = 12) and WD (*n* = 43) rats (Table S1); crosses indicate individual animals. Adjusted *p*‐values for AI_L/R_ ≠ 0 are shown. (D) Between‐group contrasts (ΔAI_L/R_) comparing WD and control rats. Plots show posterior medians (black circles), 95% highest posterior density intervals (95% HPD; black horizontal lines), and posterior distributions (colored density plots) from Bayesian regression. Effects were considered supported when the 95% HPD interval excluded zero (AI_L/R_ ≠ 0 in C; (ΔAI_L/R_) ≠ 0 in D) and the corresponding multiplicity‐adjusted *p*‐value was ≤.05 (adjusted *p*‐values are shown). Credible intervals are treated as the primary inferential output, whereas adjusted *p*‐values are reported as supplementary model‐based summaries.

The surgical procedures and time points for treatments and physiological measurements are shown in Figures 1, 2, 4, and 5. Rats were water deprived for 24 h. A rat subgroup underwent complete spinal cord transection after 24‐h WD. HL‐PA and stretching force were analyzed after 24‐h WD period (the 0‐h time point was designated as “Pre”) and then approximately 3 h later after pharmacological treatment or spinal cord transection (designated as “Post”).

SSR‐149415 and conivaptan were injected immediately after the first asymmetry analysis. SSR‐149415 was dissolved in 0.6% methylcellulose‐saline suspension and injected intraperitoneally (i.p.) at 5 mg/kg. The SSR‐149415 dose was chosen based on previous studies, in which 1–10 mg/kg i.p. doses were observed to efficiently block endocrine processes and behaviors in a variety of models.^29^, ^30^, ^31^, ^32^, ^33^ The dose 5 mg/kg, i.p. was used but not optimized due to its sufficiency to block the behavioral effects of AVP neuron activation. Conivaptan was diluted in 5% dimethyl sulfoxide (DMSO) and injected intravenously (i.v.) at 0.3 mg/kg. The dose was chosen based on previous studies, in which 0.3–3 mg/kg i.v. doses were observed to efficiently block endocrine processes and behaviors in a variety of models.^34^, ^35^, ^36^

The design of control and WD rat groups exposed to the antagonists and vehicle is shown in Figure 4A–E.

### 2.3 Spinal cord transection

The animals were anesthetized with isoflurane (1.5% isoflurane in a mixture of 65% nitrous oxide and 35% oxygen). Core temperature of the animals was controlled using a feedback‐regulated heating system. The anaesthetized animals were mounted onto the stereotaxic frame and the skin of the back was incised along the midline at the level of the upper thoracic vertebrae. After the back muscles were retracted to the sides, a laminectomy was performed at the T2 and T3 vertebrae. A 3–4‐mm spinal cord segment between the two vertebrae was dissected and removed.^21^, ^22^ The completeness of the transection was confirmed by (i) inspecting the cord during the operation to ensure that no spared spinal tissue bridged the transection site and that the rostral and caudal stumps of the spinal cord were completely retracted; and (ii) examining the spinal cord in all animals after termination of the experiment.

### 2.4 Analysis of HL‐PA

The HL‐PA value and the side of the flexed limb were assessed as described elsewhere using the hands‐off method.^21^, ^22^ Briefly, the measurements were performed under isoflurane anesthesia (1.5% isoflurane in a mixture of 65% nitrous oxide and 35% oxygen). The level of anesthesia was characterized by a barely perceptible corneal reflex and a lack of overall muscle tone. The anesthetized rat was placed in the prone position on the 1‐mm grid paper.

To measure HL‐PA, silk threads were glued to the nails of the middle three toes of each hindlimb, and their other ends were tied to one of two hooks attached to the movable platform that was operated by a micromanipulator constructed in the laboratory.^37^ To reduce potential friction between the hindlimbs and the surface with changes in their position during stretching and after releasing them, the bench under the rat was covered with a plastic sheet and the movable platform was raised up to form a 10° angle between the threads and the bench surface. The limbs were adjusted to lie symmetrically, and stretching was performed over 1.5 cm at a rate of 2 cm/s. The threads then were relaxed, the limbs were released, and the resulting HL‐PA was photographed. The procedure was repeated three times in succession, and the HL‐PA values for a given rat were used in statistical analyses.

The limb that projected over a shorter distance from the trunk was considered to be flexed. The HL‐PA was measured in mm with negative and positive HL‐PA values that were assigned to rats with the left and right hindlimb flexion, respectively. The HL‐PA was assessed by the magnitude of postural asymmetry (MPA) that shows absolute asymmetry size and by the postural asymmetry score (PAS) that shows the HL‐PA value and flexion side. The MPA does not show a flexion side. These two measures are obviously dependent; however, they are not redundant and for this reason, both are required for characterization of the HL‐PA data and presentation.

### 2.5 Analysis of hindlimb resistance to stretch

Stretching force was analyzed under isoflurane anesthesia using the micromanipulator‐controlled force meter device constructed in the laboratory.^37^ Two Mark‐10 digital force gauges (model M5‐05, Mark‐10 Corporation, USA) with a force resolution of 50 mg were fixed on a movable platform operated by a micromanipulator. Three 3–0 silk threads were glued to the nails of the middle three toes of each hindlimb, and their other ends were hooked to one of two force gauges. The flexed leg of the rat in the prone position was manually stretched to the level of the extended leg; this position was taken as the 0 mm point. Then both hind limbs were stretched caudally, moving the platform by micromanipulator at a constant rate of 5 mm/s for 10 mm. No, or very little, trunk movement was observed with stretching for the first 10 mm; therefore, the data recorded for this distance were included in statistical analysis. The forces (in grams) detected by each of the two gauges were simultaneously recorded (100 Hz frequency) during stretching. Three successive ramp‐hold‐return stretches were performed as technical replicates. Because the entire hindlimb was stretched, the measured resistance was characteristic of the passive musculo‐articular resistance integrated for hindlimb joints and muscles.^38^, ^39^, ^40^ The resistance analyzed could have both neurogenic and mechanical components, but their respective contributions were not distinguished in the experimental design. The resistance was measured as the amount of mechanical work W_L_ and W_R_ to stretch the left‐ and right hindlimbs, where W was stretching force integrated over the stretching distance interval from 0 to 10 mm.

### 2.6 Immunohistochemistry

Brains from 10 male rats (Table S1) were used for immunofluorescent staining. Rats were deprived of water supply for 24 h (WD group; *n* = 5) or provided with water ad libitum (control group; *n* = 5). Rats were deeply anaesthetized and transcardially perfused with 4% paraformaldehyde in 0.1M phosphate‐buffered saline (PBS), and their brains were postfixed in the same solution for an overnight before being transferred to 0.1M PBS containing 30% sucrose. The part of the brain that contains the hypothalamus was then coronally sectioned at 40 μm using a microtome (Microm HM450, ThermoScientific, Roskilde, Denmark). Every fourth consecutive section was used for immunofluorescent staining to identify AVP and c‐Fos expression. The sections were first incubated for 48 h at 4°C in the primary antibodies: guinea pig anti‐vasopressin (1:2000; Synaptic Systems, #403004) and mouse anti‐c‐Fos (1:500; Abcam, #ab208942), and then they were incubated in a secondary antibodies goat anti‐guinea pig Alexa Flora 594 (1:500; Invitrogen, #A11076) and goat anti‐mouse Alexa Flora 488 (1:500; Invitrogen, #A11029) for 1 h at room temperature. After washing the sections were mounted on slides and coverslipped.

Images in the aimed nuclei of the hypothalamus were captured at 20× magnification using a DM6000B Leica microscope (Leica Microsystems, Wetzlar, Germany). The colocalization of AVP‐ and c‐Fos‐immunopositive neurons was verified using confocal microscopy (Olympus FV1000 MPE, Olympus Corporation, Tokyo, Japan). Quantitative analyses were performed in 2–3 sections through the middle of the related nuclei. Positive cell numbers were counted with the help of Fiji (ImageJ 1.54 g) cell counter plugin. The average number of AVP neurons on each side in both groups was normalized to the number from the left side of the control rats and presented as relative cell number per section. The proportion of AVP and c‐Fos co‐labeled neurons in the paraventricular nucleus (PVN) and the supraoptic nucleus (SON) was calculated in relation to AVP‐immunopositive neurons. Student's *t* test was performed to test statistical significance (Sigma Plot, version 11, Softonic). Data were presented as mean ± SD. The statistically significant level was set at *p* ≤ .05.

### 2.7 Analysis of gene expression

Samples were obtained from 11 sham‐operated rats from independent experiments (Table S1). The samples were snap frozen and stored at −80°C until assay. *Avpr1a* mRNA levels were measured separately in the left and right lumbar spinal cord for each animal.

#### 2.7.1 Quantitative real‐time PCR

Total RNA was purified by using RNeasy Lipid Tissue Mini Kit (Qiagen, Valencia, CA). RNA concentrations were measured with Nanodrop (Nanodrop Technologies, Wilmington, DE). RNA (500 ng) was reverse‐transcribed to cDNA with the cDNA iScript kit (Bio‐Rad Laboratories, CA, USA) according to the manufacturer's protocol. cDNA samples were aliquoted and stored at −20°C. cDNAs were mixed with PrimePCR™ Probe assay and iTaq Universal Probes supermix (Bio‐Rad) for qPCR with a CFX384 Touch™ Real‐Time PCR Detection System (Bio‐Rad Laboratories, CA, USA) according to the manufacturer's instructions. TaqMan assay was performed in 384‐well format with TaqMan probes that are listed in Table S2.

All procedures were conducted strictly in accordance with the established guidelines for the qPCR based analysis of gene expression, consistent with the minimum information for publication of quantitative real‐time PCR experiments guidelines.^41^, ^42^ The raw qPCR data were obtained by the CFX Maestro™ Software for CFX384 Touch™ Real‐Time PCR Detection System (Bio‐Rad Laboratories, CA, USA). The *Avpr1a* mRNA levels were normalized to the geometric mean of expression levels of two reference genes, *Actb* and *Gapdh*. GeNorm software was used to analyze the gene expression stabilities (M value) of the 10 candidate reference genes (*Actb*, *B2m*, *Gapdh*, *Gusb*, *Hprt*, *Pgk*, *Ppia*, *Rplpo13a*, *Tbp*, and *Tfrc*). The calculation of the M value was based on the pairwise variation between two reference genes. If the M value was less than 1.5, it could be considered a suitable reference gene. The smaller the M value, the higher the stability of gene expression levels (https://genorm.cmgg.be/; Reference 43). The expression stability of candidate reference genes was computed for all sets of samples and identified *Actb* and *Gapdh* as the most stably expressed genes. For two regions analyzed, the gene expression stability (M values) did not exceed 0.5. The optimal number of reference genes was determined by calculating pairwise variation (V value) by the geNorm program. The V value for *Actb* and *Gapdh*, the top reference genes, was 0.12, that did not exceed the 0.15 threshold, demonstrating that analysis of these two genes is sufficient for normalization.

### 2.8 Statistical analysis

Molecular and functional asymmetry data were processed and statistically analyzed after completion of the experiments by the statisticians who were not involved in their execution. No intermediate assessment was performed to avoid any bias in data acquisition. The asymmetry data were obtained by unbiased hand‐off method and unbiased registration of stretching force.

#### 2.8.1 Analysis of body weight

Body weight was analyzed in 11 control rats (data for one rat were missing) and 43 WD rats using a generalized estimating equation (GEE) model with group, time (pre vs. post), and their interaction as fixed effects, and with repeated measurements clustered within animal. This approach accounts for the correlation between the pre and post body‐weight measurements obtained from the same rat.

#### 2.8.2 Analysis of gene expression

Lateralization was quantified per animal as an asymmetry index: AI_L/R_ = log_2_(L/R), where L and R denote the left and right side *Avpr1a* mRNA levels. Lateralization was tested by assessing whether the AI_L/R_ differed from 0 using a two‐sided Wilcoxon signed‐rank test (paired, robust to non‐normality).

#### 2.8.3 Processing of physiological data

##### Bayesian framework

Predictors and outcomes scaled and centered before we fitted Bayesian regression models via full Bayesian framework by calling *RStan 2.32.7* ^44^, ^45^ using the *brms* 2.23.0 interface.^46^ To reduce the influence of outliers, models used Student's *t* response distribution with identity link function unless explicitly stated otherwise. Models had no intercepts with indexing approach to predictors.^47^ Default priors were provided by the *brms* according to Stan recommendations.^48^ Intercepts, residual SD and group‐level SD were determined from the weakly informative prior student_t(3, 0, 10). The additional parameter ν of Student's distribution representing the degrees of freedom was obtained from the wide gamma prior gamma(2, 0.1). Group‐level effects were determined from the very weak informative prior normal(0, 10). Four MCMC chains of 40,000 iterations were simulated for each model, with a warm‐up of 20,000 runs to ensure that effective sample size for each estimated parameter exceeded 10,000^49^ producing stable estimates of 95% highest posterior density (HPD) credible intervals. MCMC diagnostics were performed according to the Stan manual. *p*‐values, adjusted using the multivariate *t* distribution with the same covariance structure as the estimates, were produced by frequentist summary in *emmeans* 2.0.1^50^ together with the medians of the posterior distribution and 95% HPD. The asymmetry and contrast between groups were defined as significant if the corresponding 95% HPD did not include zero and the adjusted *p*‐value was ≤.05. Body weight was included as a covariate.

##### HL‐PA

The magnitude of postural asymmetry (MPA), postural asymmetry score (PAS), and the proportion of asymmetric animals (P_a_) were inferred via Bayesian framework using Gaussian response distribution. Adjusted *p*‐values for MPA ≠ 0 and PAS ≠ 0, and for the contrasts (ΔMPA, ΔPAS, and ΔPa) were shown.

##### Stretching force

The amount of mechanical work W_L_ and W_R_ to stretch the left‐ and right hind limbs, respectively, was computed by integrating the smoothed stretching force measurements over stretching distance from 0 to 10 mm using loess smoothing computed by *loess* function from R package *stats* with parameters span = 0.4 and family=“symmetric.” Asymmetry was assessed both as the left/right asymmetry index AI_L/R_ = log_2_ (W_L_/W_R_). The AI_L/R_ was inferred via Bayesian framework by fitting linear multilevel models as the factor of interest.

## 3 RESULTS

### 3.1 WD effects on hindlimb posture and stretching resistance

Figures 1, 2, 4, and 5 present posterior summaries from Bayesian regression across experimental groups: posterior medians (black circles), 95% highest posterior density credible intervals (95% HPD; black horizontal lines), and posterior distributions (colored density plots). The 95% HPD interval denotes the range that contains 95% of the posterior probability mass for the parameter and can be viewed as a Bayesian analogue of a confidence interval. Asymmetry was considered supported when the 95% HPD interval excluded zero and the corresponding multiplicity‐adjusted *p*‐value was ≤.05. These adjusted *p*‐values were produced in emmeans by applying its frequentist summary to the posterior mean and covariance implied by the Bayesian model estimates, with multiple‐comparisons adjustment. We do not combine Bayesian and frequentist decision rules: credible intervals are treated as the primary inferential output, whereas adjusted *p*‐values are reported as supplementary, model‐based summaries.

We first tested whether WD can evoke HL‐PA and whether the response is directionally biased. The analysis of body weight in WD and control rats revealed a highly significant group × time interaction (*p* < 1e‐10), indicating that body weight changed differently over 24 h in the two groups (Figure S1). Control rats showed a small increase in body weight (+1.4% relative to baseline) (289 ± 72 g before vs. 293 ± 71 g after 24 h; mean change +4 ± 1 g; paired test: *p* = 2e‐06), whereas WD rats exhibited a marked decrease (−6.3% relative to baseline) (262 ± 93 g before vs. 246 ± 88 g after 24 h; mean change −17 ± 7 g; paired test: *p* < 6e‐10).

HL‐PA was quantified using three complementary readouts (Figure 1): magnitude of postural asymmetry (MPA), postural asymmetry score (PAS), and the proportion of asymmetric animals (P_a_). PAS encodes directionality (negative values indicate left hindlimb flexion; positive values indicate right hindlimb flexion). Across rats, WD produced a strong and statistically supported HL‐PA. The posterior distribution for MPA in WD rats was shifted markedly rightward relative to controls (Figure 1A), indicating substantially larger asymmetry in WD animals (supported asymmetry, 95% HPD excluding 0; adjusted *p* < 1e–10). The corresponding between‐group contrast (WD − control) for MPA was also positive and supported (Figure 1B; adjusted *p* = 2e–07), consistent with a large WD‐associated increase in asymmetry magnitude.

WD also induced a clear directional bias in the postural response. PAS values were shifted into the positive range in WD rats (Figure 1C), indicating right hindlimb flexion as the dominant effect (supported asymmetry; adjusted *p* < 1e–10). The WD − control contrast in PAS was likewise supported (Figure 1D; adjusted *p* = 5e–05), demonstrating that WD changes not only the magnitude but also the directionality of posture toward rightward flexion.

Finally, WD increased the proportion of animals exhibiting asymmetry (P_a_). The posterior distribution of P_a_ was close to zero in controls but markedly elevated in WD rats (Figure 1E; supported asymmetry; adjusted *p* < 1e–10), and the WD − control contrast in P_a_ was strongly supported (Figure 1F; adjusted *p* < 1e–10).

Body weight (Figure S1) did not correlate with the magnitude of WD‐induced PAS (Pearson *r* = .091, *p* = .56; Spearman *ρ* = 0.145, *p* = .35; *n* = 43). Together with the inclusion of body weight as a covariate in the Bayesian analyses, these results do not support body weight as a major determinant of WD‐induced HL‐PA, although minor age‐related effects cannot be excluded.

We next analyzed stretching resistance–characteristic of the passive musculo‐articular resistance integrated for hindlimb joints and muscles.^37^, ^38^, ^39^, ^40^ Although HL‐PA and hindlimb stretching resistance were measured in the same animals and reflect related aspects of asymmetric motor output, the two assessments provide complementary information on resting hindlimb posture and resistance to passive stretch, respectively. Representative recordings of passive stretching force from the left and right hindlimbs of spinalized rats are shown in Figure 2B. As expected, stretching force increased with the degree of extension. Asymmetry in stretching resistance was quantified using the asymmetry index AI_L/R_ = log_2_ (W_L_/W_R_), where W_L_ and W_R_ denote the mechanical work (g × mm) required to stretch the left and right hindlimbs, respectively. Positive AI_L/R_ values indicate greater resistance in the left hindlimb, whereas negative values indicate greater resistance in the right hindlimb. WD induced a robust and statistically supported asymmetry in stretching resistance (Figure 2C; AI_L/R_ ≠ 0 and adjusted *p* < 1e–10). In addition, AI_L/R_ differed significantly between WD and control rats, as shown by the between‐group contrast AI_L/R_ (Figure 2D; ΔAI_L/R_ ≠ 0 and adjusted *p* = 9e–06). Importantly, the WD‐evoked effect was side‐specific: in WD rats, the right hindlimb exhibited greater resistance to stretch than the left.

Together, the HL‐PA and stretching force measures converge on the conclusion that WD induces a robust, population‐level asymmetric phenotype characterized by a right‐biased directional response.

### 3.2 Activation of AVP neurons by WD: Effects on c‐Fos

**FIGURE 3:**
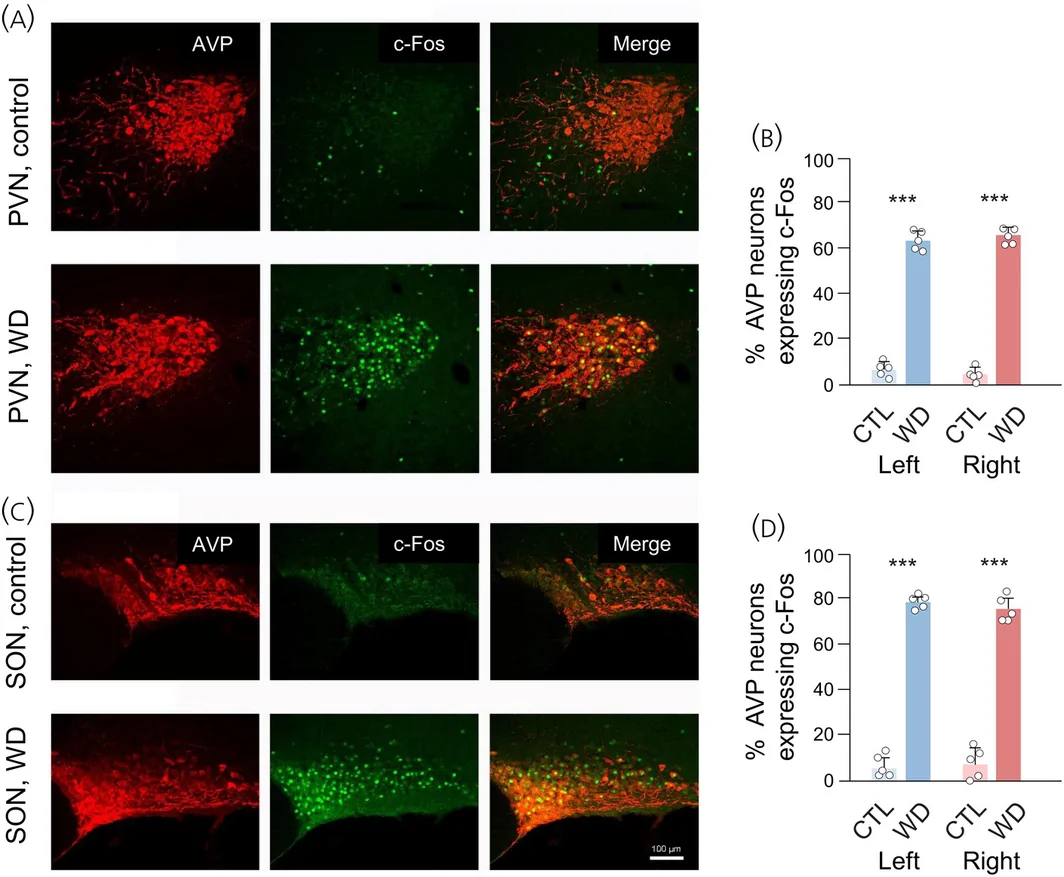
WD activates AVP neurons in the paraventricular (PVN) and supraoptic (SON) nuclei, as revealed by c‐Fos expression. (A, C) Representative images showing co‐localization of AVP immunoreactivity and c‐Fos immunoreactivity (a marker of neuronal activation) in neurons of the right PVN and right SON in control and WD rats. (B, D) Proportion of AVP‐immunopositive neurons expressing c‐Fos in the left and right PVN and SON of control (CTL) and WD rats. Data are presented as mean ± SD for five male rats per group; circles indicate individual animals. Statistical significance was assessed using a two‐tailed unpaired *t* test. ***p < .001. Scale bar = 100 μm.

Because dehydration recruits the hypothalamic–neurohypophysial AVP system,^26^, ^27^, ^28^ we next examined whether WD activates hypothalamic AVP neurons. AVP‐immunopositive neurons were detected in the magnocellular division of the PVN, SON, and suprachiasmatic nucleus (SCN) (Figure 3). To assess AVP‐neuron activation, we quantified the proportion of c‐Fos‐immunopositive cells among AVP neurons in the PVN and SON.

WD induced a marked increase in c‐Fos expression in AVP neurons in both nuclei. Averaged across the left and right sides, 64.2 ± 3.5% of AVP neurons in the PVN and 77.7 ± 3.9% in the SON were c‐Fos‐positive in WD rats, compared with 5.6 ± 2.6% and 7.4 ± 5.9%, respectively, in control rats (Figure 3). The proportion of c‐Fos‐positive AVP neurons was significantly higher in WD than in control animals in both the PVN and SON (*p* < .001 for both nuclei, two‐tailed unpaired *t* test), indicating robust activation of hypothalamic AVP neurons by WD. The SCN was not analyzed quantitatively because individual AVP‐positive cell bodies were difficult to resolve reliably. No gross left–right differences in AVP immunostaining patterns were evident in the PVN or SON in either control or WD rats, consistent with our previous observations.^51^ Thus, WD activates a large proportion of AVP neurons in the hypothalamic PVN and SON.

### 3.3 Effects of AVP receptor antagonists and spinal cord transection on WD‐induced asymmetric effects

**FIGURE 4:**
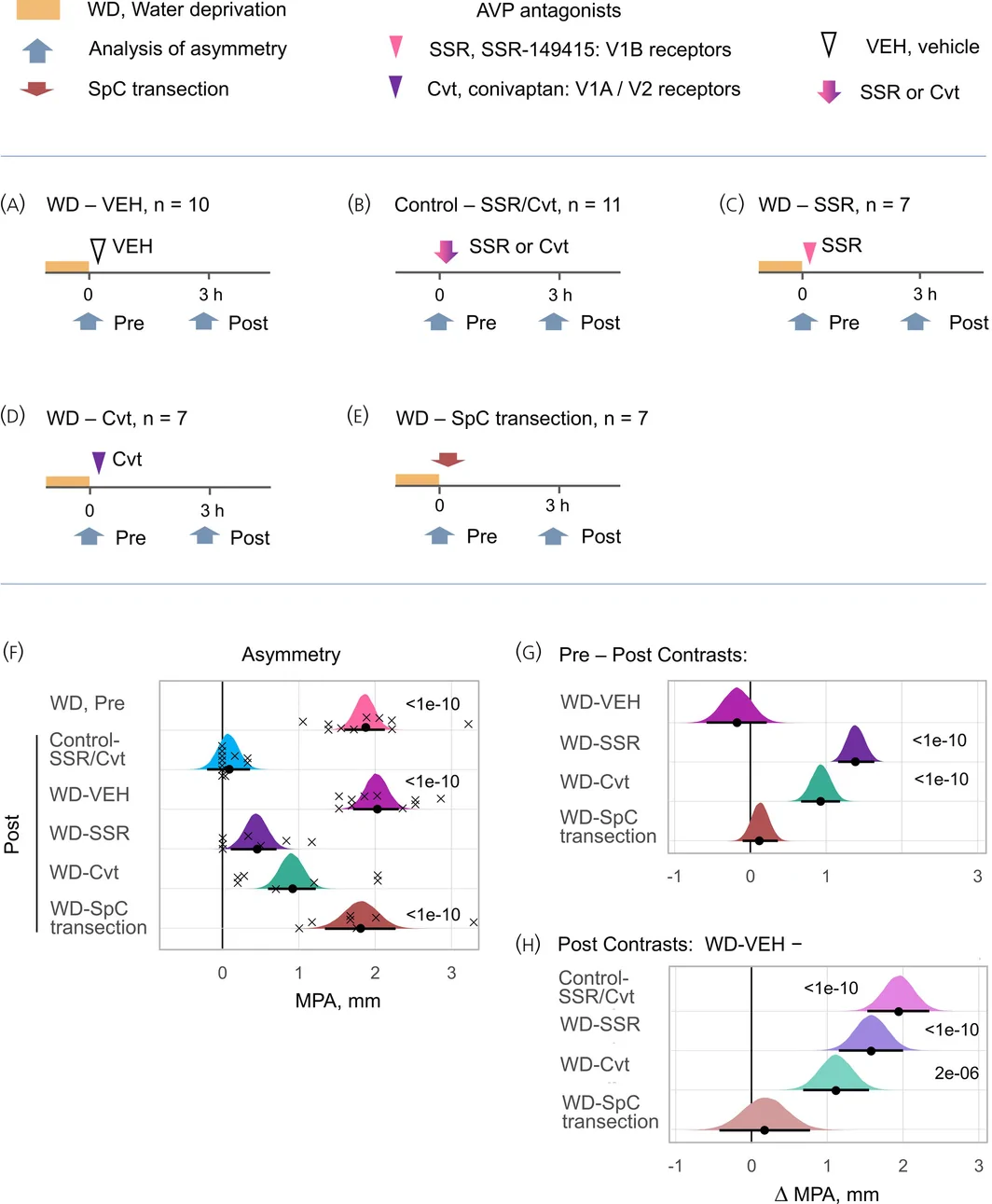
Effects of V1B and V1A/V2 receptor antagonism on WD–induced HL‐PA and its persistence after spinal cord transection. WD induced HL‐PA, quantified as the magnitude of postural asymmetry (MPA). The effects of SSR‐149415 (SSR; V1B antagonist) and conivaptan (Cvt; V1A/V2 antagonist) were tested, as well as the persistence of WD‐induced HL‐PA after complete spinal cord transection. (A–E) Experimental design for five groups comprising WD and control rats. Procedures included: (i) two assessment time points; (ii) administration of vehicle (VEH; saline with DMSO or methylcellulose–saline suspension), SSR, or Cvt; and/or (iii) complete spinal cord transection at T2–T3. In WD groups, asymmetry was first measured after 24 h WD (Pre, baseline) (A, C–E); in control rats, baseline was measured under control conditions (B). Treatments were then administered and/or the spinal cord transected, and HL‐PA was re‐assessed 2–3 h later (Post). (A) The vehicle‐treated WD group consisted of 7 rats receiving 2% DMSO in saline and 3 rats receiving 0.6% methylcellulose in saline after the first asymmetry analysis (Pre). These subgroups did not differ significantly in MPA but did not satisfy predefined conservative equivalence criteria. Therefore, the pooled vehicle analysis is presented only as a secondary descriptive comparison and is not used to support the principal conclusions of the study. (B) The control group treated after the first asymmetry analysis (Pre) consisted of 4 rats receiving SSR‐149415 and 7 rats receiving conivaptan. Formal equivalence analysis supported pooling of these subgroups. (F) MPA values for WD rats at Pre and Post time points, and for control rats at the Post time point; crosses indicate individual animals (WD rats show MPA > 1 mm). (G) Within‐group contrasts in MPA for WD groups, defined as ΔMPA = MPA(Post) − MPA(Pre). (H) Between‐group contrasts at the Post time point comparing the WD‐vehicle group (reference group with robust asymmetry) to each test group, defined as ΔMPA = MPA(WD‐VEH) − MPA(test group). Posterior summaries from Bayesian regression are shown as posterior medians (black circles), 95% highest posterior density (HPD) intervals (black horizontal lines), and posterior distributions (colored density plots). Effects were considered supported when the 95% HPD interval excluded zero and the corresponding multiplicity‐adjusted *p*‐value was ≤.05 (adjusted *p*‐values are shown). Credible intervals are the primary inferential summaries; adjusted *p*‐values are reported as supplementary model‐based estimates.

To probe the mechanisms underlying WD‐evoked HL‐PA, we next tested whether the response depends on AVP receptor signaling and whether it persists after disruption of descending spinal pathways. Five experimental designs were implemented (Figure 4A–E and Table S1): WD animals received vehicle (WD–VEH; *n* = 10), the V1B antagonist SSR‐149415 (WD–SSR; *n* = 7), or the V1A/V2 antagonist conivaptan (WD–Cvt; *n* = 7); a separate WD group underwent complete spinal cord transection (WD–SpC; *n* = 7); and control animals were tested under antagonist exposure (Control–SSR/Cvt; *n* = 11). HL‐PA was assessed at baseline after 24 h WD (Pre) and again ~3 h after intervention (Post).

WD animals already displayed pronounced asymmetry at baseline (Pre) (Figure 4F; WD Pre), consistent with the robust WD phenotype in Figure 1. Control rats treated with antagonists (Control–SSR/Cvt) showed low MPA at the Post time point (Figure 4F), indicating that antagonist exposure per se did not induce HL‐PA.

In vehicle‐treated WD animals, MPA remained high at the Post time point (Figure 4F; WD–VEH), showing persistence of the WD phenotype over the measurement interval. When SSR‐149415, a selective V1B receptor antagonist, was administered after WD, Post MPA was markedly reduced (Figure 4F; WD–SSR). This reduction was supported by the within‐group Pre–Post contrast (Figure 4G; supported change with adjusted *p* < 1e–10) and by the between‐group contrast relative to WD–VEH at Post (Figure 4H; WD–VEH − WD–SSR; supported difference with adjusted *p* < 1e–10).

Similarly, administration of conivaptan (V1A/V2 antagonist) strongly reduced WD‐evoked MPA (Figure 4F; WD–Cvt). The within‐group Pre–Post contrast indicated a supported reduction (Figure 4G; adjusted *p* < 1e–10), and the Post contrast relative to WD–VEH was also supported (Figure 4H; adjusted *p* = 2e–06). Thus, V1B signaling and V1A and/or V2 signaling are required for full expression of the WD‐evoked HL‐PA phenotype.

**FIGURE 5:**
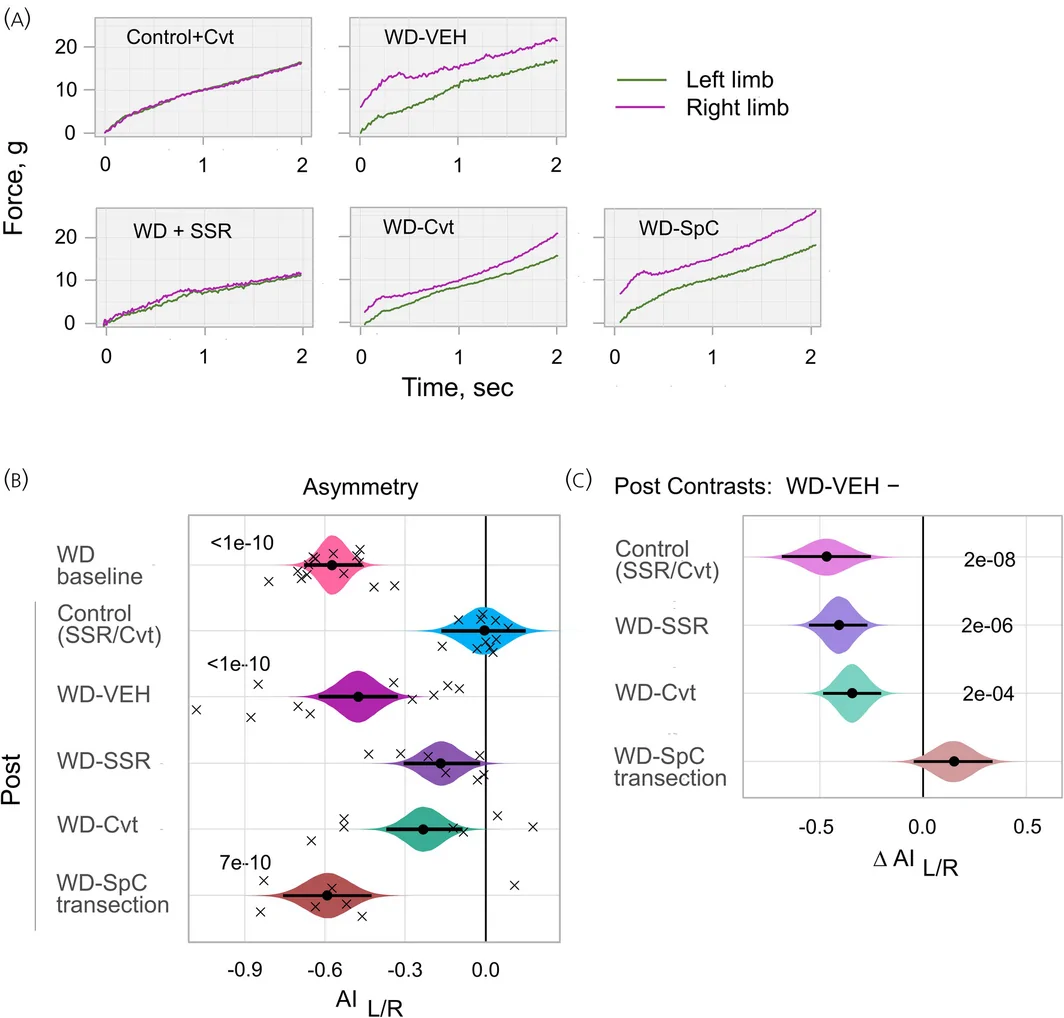
Effects of V1B and V1A/V2 receptor antagonism and spinal cord transection on WD–induced asymmetry in hindlimb stretching resistance. WD‐induced asymmetry in hindlimb stretching resistance was quantified as AI_L/R_ = log_2_(W_L_/W_R_). The effects of SSR‐149415 (SSR; V1B antagonist) and conivaptan (Cvt; V1A/V2 antagonist) were tested, as well as the persistence of WD‐induced asymmetry after complete spinal cord transection. The design of the five groups, treatment protocols, and animal numbers are detailed in Figure 4A–E and Table S1. Procedures included: (i) administration of vehicle (saline with DMSO or methylcellulose–saline suspension), SSR‐149415, or conivaptan and/or (ii) complete spinal cord transection at T2–T3. In the control group, rats received SSR‐149415 (*n* = 4) or conivaptan (*n* = 7). In the WD‐VEH group, 7 rats received 2% DMSO in saline and 3 rats received 0.6% methylcellulose–saline. Formal equivalence analysis supported the pooling of their subgroups within each of the control group and the vehicle‐treated WD group. The SSR‐149415, conivaptan, and spinal transection groups each contained 7 rats. Stretching force was assessed at baseline and again 2–3 h after treatment (Pre and Post, respectively). Treatment effects on AI_L/R_ were evaluated within each group, and between‐group contrasts were assessed at the Post time point. (A) Representative force traces recorded from the left and right hindlimbs. (B) Post‐treatment AI_L/R_ values for the five groups; baseline AI_L/R_ values for WD rats are shown for comparison. Crosses indicate individual animals. (C) Between‐group contrasts comparing the WD‐VEH group (reference group with robust asymmetry) with the other groups at 2–3 h Post: ΔAI_L/R_ = AI_L/R_ (WD‐VEH) − AI_L/R_ (test group). Plots show posterior medians (black circles), 95% highest posterior density intervals (95% HPD; black horizontal lines), and posterior distributions (colored density plots) from Bayesian regression. Effects were considered supported when the 95% HPD interval excluded zero (AI_L/R_ ≠ 0 in B; ΔAI_L/R_ ≠ 0 in C) and the corresponding multiplicity‐adjusted *p*‐value was ≤.05 (adjusted *p*‐values are shown). Credible intervals are treated as the primary inferential summaries, whereas adjusted *p*‐values are reported as supplementary model‐based estimates.

Consistent with the HL‐PA results, blockade of V1B receptors with SSR‐149415 and blockade of V1A/V2 receptors with conivaptan markedly reduced the WD‐induced asymmetry in stretching resistance (Figure 5A,B). These effects were supported by the between‐group contrasts at the Post time point relative to the WD‐VEH group (Figure 5C; WD–VEH − WD–SSR and WD–VEH − WD–Cvt). Thus, V1B signaling and V1A and/or V2 signaling contribute to the expression of WD‐evoked asymmetry in hindlimb stretching resistance.

Finally, both WD‐induced HL‐PA and asymmetry in stretching resistance persisted after complete thoracic spinal cord transection. In the transected WD group (WD–SpC), post‐treatment MPA and AI_L/R_ remained elevated and showed no meaningful reduction relative to the WD–VEH group at the Post time point (Figures 4G and 5C; the WD–VEH − WD–SpC contrast was centered near zero). This persistence indicates that expression of the WD‐evoked asymmetry does not require intact descending spinal pathways.

Together, these results show that WD‐produced asymmetric effects are significantly reduced by V1B antagonism and V1A/V2 antagonism, and maintained after complete spinal cord transection, supporting the involvement of endocrine/humoral signaling in generating the asymmetric postural and sensorimotor responses.

### 3.4 *Avpr1a* expression in the left and right lumbar spinal cord

**FIGURE 6:**
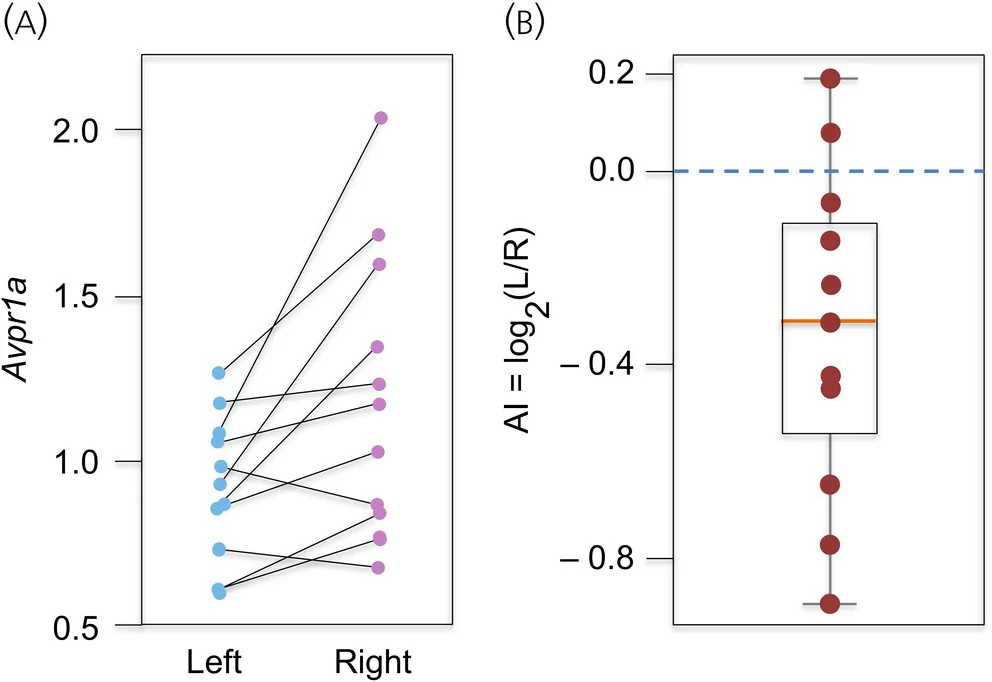
Right‐biased expression of *Avpr1a* in the rat spinal cord. (A) Paired left–right *Avpr1a* expression values for each animal (one line per rat). (B) Data are presented as an asymmetry index AI = log_2_(L/R), where L and R denote the left and right side *Avpr1a* mRNA levels. AI <0 indicates right‐biased expression and AI >0 indicates left‐biased expression. Individual data points are shown together with box‐and‐whisker plots (median, interquartile range, minimum and maximum values). Right‐biased lateralization was assessed by testing whether the asymmetry index differed from zero (Wilcoxon signed‐rank test, *p* = .0098). Samples were obtained from 11 sham‐operated rats from independent experiments.

Because the WD phenotype is directionally biased (right hindlimb flexion) and is sensitive to antagonism of AVP receptors, we examined whether components of AVP signaling in the lumbar spinal cord are themselves laterally organized. We measured *Avpr1a* mRNA in left and right lumbar spinal samples (Figure 6; *n* = 11 rats).

Paired within‐animal comparisons revealed a consistent rightward bias: *Avpr1a* expression was higher on the right than on the left in the majority of animals (Figure 6A). This lateralization was confirmed by testing the asymmetry index (AI_L/R_ = log_2_(L/R), where L and R denote the left and right side *Avpr1a* mRNA levels) against zero (Figure 6B; paired Wilcoxon signed‐rank test, *p* = .0098). Thus, *Avpr1a* expression in lumbar spinal cord is laterally organized, providing a plausible “decoder” substrate through which circulating AVP‐dependent signals could be translated into side‐specific motor output.

## 4 DISCUSSION

The first finding of this study is that WD induces HL‐PA characterized by right hindlimb flexion. This effect persisted after complete spinal transection suggesting that the asymmetry is maintained through humoral signaling but not commands descending from the brain to the spinal cord through the neural tracts. WD is a symmetric homeostatic challenge that engages the lamina terminalis–hypothalamic osmoregulatory network and the AVP system, including the subfornical organ/organum vasculosum lamina terminalis‐driven thirst and feedforward/feedback regulation of AVP secretion.^52^, ^53^ WD and water restriction also recruit endocrine stress‐effector pathways mediated by the pro‐opiomelanocortin (POMC)‐derived adrenocorticotropic hormone and β‐endorphin, an endogenous opioid peptide.^54^, ^55^ In parallel, WD recruits PVN “pre‐autonomic” control of sympathetic outflow via PVN projections to the spinal cord and brainstem (e.g., the rostral ventrolateral medulla), providing an anatomical route by which a symmetric internal state can be translated into coordinated neuroendocrine–autonomic output.^56^, ^57^ No evidence, as far as we know, has been presented that WD by itself can drive left‐ or right‐side dominant PVN program. Thus, a conservative interpretation of our findings—the WD lateralization effects—is that HL‐PA and its direction (right hindlimb flexion) are set downstream of a symmetric neuroendocrine activation rather than by lateralized PVN recruitment. This hypothesis is supported by our observation that symmetric chemogenetic stimulation of the hypothalamic AVP circuit in transgenic rats produces HL‐PA with right hindlimb flexion.^51^

Our WD findings corroborate a previous study showing that symmetric challenge—cold and immobilization stress as well as visceral pain—also produces asymmetric response, HL‐PA in rats.^58^ The postural effects were evident after complete spinal cord transection and left limb flexion predominated as a trend. The effects were apparently mediated via opioid receptors—the opioid antagonist naloxone blocked the HL‐PA.

### 4.1 The AVP mechanism underlying WD‐induced HL‐PA

The present findings support the conclusion that neuroendocrine AVP signaling contributes to WD‐induced HL‐PA. This interpretation is supported by two observations: (i) antagonists of AVP receptors markedly reduced WD‐induced HL‐PA and (ii) AVP itself, administered symmetrically (intravenously or intracisternally), as well as chemogenetic activation of hypothalamic AVP neurons, elicits HL‐PA with right hindlimb flexion.^21^, ^51^ In these previous experiments, the effects of exogenous AVP and activation of AVP neurons were observed in rats after complete spinal cord transection, demonstrating that they can be transmitted through humoral pathways independently of descending neural tracts. Together, these findings identify AVP signaling as a necessary component of WD‐induced HL‐PA but do not establish the complete neuroendocrine pathway linking dehydration to asymmetric spinal motor output.

**FIGURE 7:**
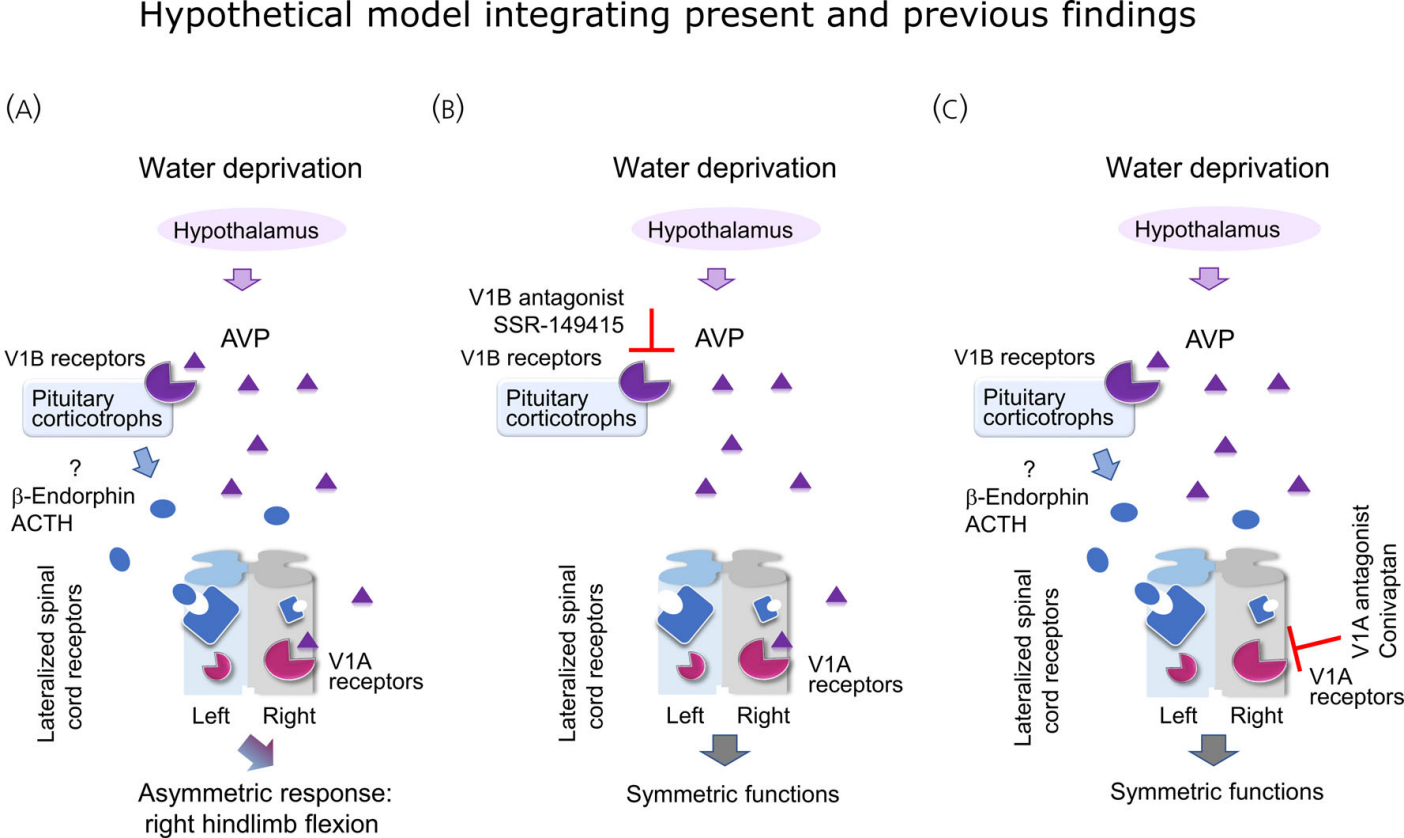
Proposed hypothetical two‐site (“two‐hit”) working model. The model integrates the present pharmacological findings with previous experimental evidence and is intended as a framework for future experimental testing rather than an experimentally established pathway. (A) The observation that both the V1B antagonist SSR‐149415 and the V1A/V2 antagonist conivaptan suppress WD‐induced HL‐PA is consistent with a model in which circulating AVP released from magnocellular hypothalamic neurons acts at two functionally distinct targets: (1) a proximal pituitary site that may contribute to the asymmetric state through endocrine signaling, and (2) a distal spinal site that modulates sensorimotor excitability. One candidate proximal mechanism involves V1B receptors expressed predominantly in corticotrophs, through which AVP facilitates secretion of POMC‐derived hormones, including ACTH and β‐endorphin.^65^ One or more circulating POMC‐derived hormones could subsequently contribute to formation of HL‐PA, as previously demonstrated for β‐endorphin.^21^ The distal “spinal hit” may involve V1A receptors within ventral horn sensorimotor circuitry, where AVP modulates neuronal excitability and synaptic transmission,^59^, ^60^, ^61^ consistent with the high density of vasopressin binding sites in spinal motor nuclei.^62^ We further hypothesize that left–right specificity is determined by lateralized spinal “decoder” mechanisms: *Avpr1a* (V1A receptor) expression is right‐biased in the spinal cord (present study), whereas opioid receptor gene expression exhibits left‐biased expression patterns (References 20, 22, 23, and references therein). Together, these asymmetrically expressed receptor systems could enable circulating AVP and one or more POMC‐derived hormones to generate side‐specific motor output by differentially biasing mirror‐symmetric spinal circuits. (B, C) Within this working model, antagonism at either site is predicted to suppress the asymmetric state. V1B receptor blockade would be expected to reduce AVP‐dependent pituitary release of POMC‐derived hormones, whereas V1A/V2 receptor blockade would be expected to reduce AVP actions on spinal sensorimotor circuitry. In both cases, asymmetric motoneuron drive would be reduced, resulting in restoration of postural symmetry. This model predicts that AVP signaling and pituitary‐derived hormone(s) together contribute to WD‐induced left–right symmetry breaking; however, the identity of the circulating mediator(s) downstream of pituitary V1B activation remains unknown and was not investigated in the present study.

One possible interpretation of the present findings is a hypothetical two‐site (“two‐hit”) AVP model, in which circulating AVP released from magnocellular hypothalamic neurons engages both a distal spinal target and a proximal pituitary target. Figure 7 summarizes this working hypothesis rather than an experimentally established pathway.

The pharmacology identifies the necessity of AVP signaling but not all anatomical sites involved. One candidate distal site is the spinal cord, where V1A receptors may mediate AVP‐dependent modulation of sensorimotor circuitry.^59^, ^60^, ^61^ AVP directly depolarizes identified motoneurons and facilitates inhibitory transmission in ventral horn neurons, effects mediated predominantly via V1A rather than V1B receptors. Consistently, AVP binding sites are present at high density in somatic motor nuclei of the spinal cord.^62^ In addition, AVP signaling influences spinal sensory processing and plasticity in pain pathways,^63^, ^64^ supporting the capacity of spinal V1A signaling to reshape sensorimotor network excitability.

The side‐specificity of WD‐induced HL‐PA suggests that circulating AVP differentially biases mirror‐symmetric spinal circuits controlling left and right hindlimb muscles, potentially through asymmetrically distributed AVP receptors. Consistent with this interpretation, *Avpr1a* expression is right‐biased in the spinal cord (this study). One candidate proximal site is the pituitary gland, where V1B receptors, expressed predominantly in corticotrophs, may potentiate AVP‐dependent release of POMC‐derived hormones, including ACTH and β‐endorphin^65^ (Figure 7). One or more circulating POMC‐derived peptides could subsequently contribute to formation of right‐flexion HL‐PA, as previously demonstrated for β‐endorphin.^21^ However, neither pituitary hormone release nor the contribution of individual POMC‐derived peptides was examined in the present study. Consequently, this proposed pathway should be regarded as hypothetical and intended primarily as a framework for future experimental testing.

The efficacy of SSR‐149415 and conivaptan in inhibiting asymmetry was apparently different. However, this should be interpreted with caution. Although SSR‐149415 abolished HL‐PA whereas conivaptan produced only partial attenuation under the present experimental conditions, the two antagonists were administered at different doses, by different routes (intraperitoneal versus intravenous), and in different vehicles. Consequently, differences in the magnitude of their effects may reflect pharmacological factors, including bioavailability, tissue distribution, or pharmacokinetics, rather than a mechanistic distinction between V1B‐dependent pituitary signaling and V1A‐dependent spinal signaling. Direct comparison of the relative contributions of these pathways will require experiments using matched pharmacological protocols and dose–response analyses.

Conivaptan was administered intravenously and therefore blocked peripheral V1A/V2 receptors systemically. Consequently, although the right‐biased expression of spinal *Avpr1a* provides a plausible anatomical substrate for asymmetric AVP actions, the present experiments do not establish the spinal cord as the exclusive site of V1A‐mediated action. Additional contributions from peripheral tissues or circumventricular brain regions lacking a functional blood–brain barrier cannot be excluded. Thus, the proposed spinal component of the two‐site model should be regarded as one mechanistically plausible interpretation that is strongly supported by but not conclusively established by the present findings. Further experiments using region‐specific pharmacological or genetic approaches are necessary to determine the relative contributions of spinal and supraspinal V1A signaling.

Previous studies demonstrated that AVP induces side‐specific postural effects in rats whose descending neural control of spinal circuits had been abolished by complete spinal transection.^21^, ^51^ In contrast, HL‐PA develops here in WD animals with an intact spinal cord. These findings raise the possibility that WD reduces the influence of descending supraspinal pathways, thereby allowing asymmetric neuroendocrine signaling to become functionally expressed. This possibility remains to be investigated experimentally.

HL‐PA was assessed approximately 3 h after complete thoracic spinal cord transection, a period that may overlap with the acute phase of spinal shock. Several observations argue against passive persistence of a pre‐existing asymmetric posture. First, in the present study, both SSR‐149415 and conivaptan reversed WD‐induced HL‐PA within a comparable post‐transection interval, indicating that the asymmetry requires active neurohumoral maintenance. Second, previous studies showed that HL‐PA induced by unilateral brain injury or by neurohormones, including AVP and opioid peptides, is evident shortly after spinal transection and persists in spinalized preparations for at least 4–5 h.^21^, ^22^, ^23^, ^24^, ^37^ Thus, although acute spinal shock may modulate spinal excitability and thereby influence the magnitude of the phenotype, it is unlikely to account for WD‐induced HL‐PA. Nevertheless, the possibility that spinal shock interacts with neurohumoral signaling to influence the expression of HL‐PA cannot be excluded. The spinal mechanisms through which humoral AVP signaling is converted into asymmetric hindlimb posture remain unresolved. Segmental proprioceptive circuits that survive transection and regulate muscle tone may contribute, as may asymmetric tonic activity of spinal motoneurons or spinal interneuronal networks.

### 4.2 Why does WD induce HL‐PA?

One interpretation of the WD phenotype is that dehydration recruits the AVP system as part of the homeostatic response to thirst.^66^ While activation of neuroendocrine pathways represents a common feature of homeostatic challenges, it remains unclear why this response should produce a directional motor asymmetry. Furthermore, the opposite directions of HL‐PA induced by WD versus previously reported cold stress and pain^58^ suggest that these responses are not stereotyped but depend on the modality of the homeostatic challenge. Within this framework, AVP may represent one example of a neuroendocrine signal capable of producing a right‐biased motor output, whereas other neuroendocrine pathways recruited by cold stress or pain may generate the opposite directional bias.

Beyond the present experimental findings, one broader speculative implication deserves consideration. The binary directions of HL‐PA induced by WD, cold stress, and pain are consistent with the previously proposed bipartite organization of the topographical‐neuroendocrine system, in which the left and right hemispheres communicate with the body through distinct neuroendocrine pathways.^21^, ^22^ The WD‐induced directional asymmetry is also compatible with previous observations that interoceptive and autonomic functions exhibit functional lateralization, including cortical processing of thirst, visceral sensory information, and asymmetric autonomic responses during emotionally salient situations.^6^, ^51^, ^66^, ^67^, ^68^, ^69^, ^70^, ^71^ Together, these observations suggest that physiological regulation may become lateralized to the left or the right even when the initiating homeostatic challenge is itself symmetric.

If such a broader organizational principle exists, HL‐PA may provide an experimentally accessible motor readout of transient shifts in bilateral neuroendocrine regulation rather than representing an adaptive endpoint itself. Whether different homeostatic challenges recruit distinct neuroendocrine pathways that differentially regulate the balance between left‐ and right‐sided body functions, thereby generating opposite directions of lateralized motor output, remains to be determined.

### 4.3 Limitations

This study has several limitations. First, we assessed WD–evoked lateralization using the HL‐PA model under anesthesia. This approach provides a robust and standardized readout of side‐specific output and helps minimize supraspinal contributions, thereby isolating a neuroendocrine component. However, it does not capture the full spectrum of posture and motor coordination in awake animals. In freely behaving WD rats, voluntary and reflex postural corrections may compensate for an endocrine‐driven bias, whereas reduced descending control under anesthesia may unmask it as persistent asymmetry. Future studies should therefore test whether WD induces measurable lateralized motor bias in awake animals using complementary behavioral paradigms, which would strengthen translational relevance. Second, although WD produced lateralized effects, the underlying neuroendocrine mechanism remains incompletely resolved. The anatomical sites at which AVP initiates and maintains the response—and the principal loci of antagonist action (hypothalamic, pituitary, spinal, vascular, or peripheral)—cannot be determined from the present design. Third, it remains unclear whether the relevant humoral signal is elevated AVP alone, or a shift in the balance of circulating factors that bias left–right output, with AVP being one component (e.g., involvement of POMC‐derived peptides such as β‐endorphin). To test the “multiple circulating signals” hypothesis directly, future work should profile the plasma neuropeptidome after WD—including AVP and POMC‐derived peptides (e.g., ACTH, β‐endorphin/β‐LPH, and MSH)—and determine whether candidate factors exert side‐specific activity. Additional limitations include reliance on a single V1B antagonist and a single V1A/V2 antagonist. While SSR‐149415 is widely used as a V1B antagonist in rodents,^29^, ^30^, ^31^, ^32^, ^33^, ^71^, ^72^ pharmacological specificity cannot be fully established without antagonist replication and site‐restricted manipulations. Moreover, blockade by conivaptan is consistent with a spinal AVP component, but because conivaptan also targets V2 receptors and can influence systemic fluid balance, resolving V1A‐ versus V2‐dependent contributions will require a V1A‐selective antagonist and/or spinally localized manipulations.

Despite these constraints, the present findings provide a foundation for mechanistic dissection of hypothalamic control of lateralized peripheral physiology at circuit and behavioral levels under physiological conditions.

## 5 CONCLUSIONS

WD‐induced HL‐PA can be viewed as a functional readout of neuroendocrine symmetry breaking, in which AVP‐linked hormonal states can determine a directional peripheral motor response under symmetric stimulation. WD is a spatially symmetric systemic challenge, yet it produces a population‐level directional HL‐PA characterized by right hindlimb flexion. Thus, WD appears to recruit an internal symmetry‐breaking mechanism that converts a non‐directional homeostatic state into directional postural “set‐point” (“symmetric challenge → asymmetric response”). AVP signaling—acting via V1B and V1A‐dependent mechanisms—may initiate and maintain (“lock”) this set‐point once formed. The findings raise questions on (i) how general the phenomenon of side‐specific processing of external symmetric stimuli and left–right‐sided response is to them, and on (ii) the side‐specific neurohormonal regulation of paired organs and tissues as a driving force of such responses in the bilaterians.

## AUTHOR CONTRIBUTIONS

**Yaromir Kobikov:** Data curation; formal analysis; investigation; software. **Alfhild Grönbladh:** Formal analysis; investigation. **Jens Schouenborg:** Resources; supervision. **Olga Nosova:** Formal analysis; investigation. **Georgy Bakalkin:** Conceptualization; writing – original draft; supervision; project administration; writing – review and editing; resources; funding acquisition. **Sara Yusuf Mohamed:** Data curation; formal analysis; investigation. **Mathias Hallberg:** Formal analysis; investigation; resources. **Mengliang Zhang:** Conceptualization; writing – original draft; investigation; supervision; project administration; writing – review and editing; resources; funding acquisition. **Hiroyuki Watanabe:** Formal analysis; methodology; investigation. **Daniil Sarkisyan:** Data curation; investigation; software. **Karen Rich:** Formal analysis.

## FUNDING INFORMATION

The study was supported by the Swedish Research Council (Grant 2022‐01182) to G.B., and Novo Nordisk Foundation (NNF20OC0065099) to M.Z.

## CONFLICT OF INTEREST STATEMENT

The authors declare no conflicts of interest.

## Supporting information


**Figure S1.** Effects of 24 h water deprivation (WD) on body weight. Body weight was measured before (Pre) and after 24 h of WD (Post), or on the same days in control rats. Data are shown as box plots with superimposed individual values for Control (*n* = 11) and WD (*n* = 43) groups. Boxes indicate the interquartile range, horizontal lines indicate the median, whiskers extend to 1.5 × IQR, and circles represent individual rats. Body weight changed differentially over time between groups, as assessed by a generalized estimating equation (GEE) model for repeated measures (group × time interaction, *p* < 1e‐10). Control rats showed a small increase in body weight, whereas WD rats exhibited a marked decrease.
**Table S1.** The number of rats used in the study.
**Table S2.** PCR probes (Bio‐Rad Laboratories, CA, USA) used for analysis of the *Avpr1a* gene expression.

## Data Availability

The data that support the findings of this study are available on request from the corresponding author. Data supporting the findings of this study and all codes used for analysis are available within the article, its Supporting Information and on https://github.com/YaromirKo/biostatistics-wd.
