# Supplementary material for: A symmetric systemic challenge elicits a right‐biased response mediated by vasopressin signaling
Source: J Neuroendocrinol. 2026 Sep 6;38(9):e70257. doi: 10.1111/jne.70257 (PMC13547646; doi:10.1111/jne.70257)
Supplement: Supplementary file 1 [file JNE-38-e70257-s001.docx]

**Supplementary material**

**A Symmetric Systemic Challenge Elicits a Right-Biased Response Mediated by Vasopressin Signaling**

Hiroyuki Watanabe, Yaromir Kobikov, Sara Yusuf Mohamed, Karen Rich, Daniil Sarkisyan, Olga Nosova, Alfhild Grönbladh, Mathias Hallberg, Jens Schouenborg, Georgy Bakalkin, and Mengliang Zhang

**Figure S1. Effects of 24 h water deprivation (WD) on body weight**. Body weight was measured before (Pre) and after 24 h of WD (Post), or on the same days in control rats. Data are shown as box plots with superimposed individual values for Control (n = 11) and WD (n = 43) groups. Boxes indicate the interquartile range, horizontal lines indicate the median, whiskers extend to 1.5 × IQR, and circles represent individual rats. Body weight changed differentially over time between groups, as assessed by a generalized estimating equation (GEE) model for repeated measures (group × time interaction, P < 1e-10). Control rats showed a small increase in body weight, whereas WD rats exhibited a marked decrease.

**Table S1.** The number of rats used in the study.

WD, water deprived; BW, pre-experimental body weights range, g.

| Figures 1 and 2  rat groups | n | BW, g |
| --- | --- | --- |
| Control | 11 (12*) | 185–392 |
| WD | 43 | 147–474 |

* Eleven control rats were used in both the HL-PA and stretching resistance analyses. One additional rat was analyzed for stretching resistance only at the pre-time point but was then excluded due to technical reasons.

| Figures 4 and 5 rat groups | Description | n | BW, g |
| --- | --- | --- | --- |
| WD | Pre time point: Figures 4 and 5  Pre time point: Figure 5 | 10  14 |  |
| WD–VEH | Vehicle after WD (7 DMSO-saline + 3 methylcellulose-saline; pooled) | 10 | 199–474 |
| Control–(SSR/Cvt) | Control rats treated with SSR-149415 (4) or conivaptan (7); pooled | 11 | 218–392 |
| WD–SSR | WD rats treated with SSR-149415 | 7 | 147–209 |
| WD–Cvt | WD rats treated with conivaptan | 7 | 149–288 |
| WD–SpC | WD rats with complete T2–T3 transection | 7 | 311–412 |

| Figure 3 | n | BW, g |
| --- | --- | --- |
| Control | 5 | 162-200 |
| WD | 5 | 153-191 |

| Figure 6 | n | BW, g | |
| --- | --- | --- | --- |
| Sham | 11 | 150-200 | |
|  |  |  |  |

**Table S2.** PCR probes (Bio-Rad Laboratories, CA, USA) used for analysis of the *Avpr1a* gene expression.

| **Gene symbol** | **Gene name** | **Assay ID** | **Channel** |
| --- | --- | --- | --- |
| *Actb* | Actin beta | qRnoCIP0050804 | FAM |
| *Avpr1a* | Arginine Vasopressin | qRnoCEP0023750 | FAM |
| *Gapdh* | Glyceraldehyde-3-phosphate dehydrogenase | qRnoCIP0050838 | FAM |

**Resources table**

| Reagent type  (species) or resource | Designation | Source or reference | Identifiers | Additional  information |
| --- | --- | --- | --- | --- |
| Strain, Strain  background (*Rattus norvegicus*) | Wistar Hannover rat, males | Janvier-labs, France |  |  |
| Chemical compound, drug | Isoflurane | Abbott Laboratories, Norway | NDC 0044-5260-03 | Anesthesia agent |
| Commercial  Assay or kit | RNasy Lipid Tissue Mini Kit | Qiagen, CA, USA | Cat#: 74136 | Total RNA extraction |
| Commercial  Assay or kit | iScript ™ cDNA Synthesis Kit | Bio-Rad Laboratories, USA | Cat#: 1708891 | cDNA Synthesis |
| Commercial  Assay or kit | iTaq Universal Probes supermix | Bio-Rad Laboratories, USA | Cat# 1725131 | Real-Time PCR reagent |
| Software, algorithm | RStan |  | 2.32.7 | Bayesian model fitting / R interface to Stan |
| Software, algorithm | R program | R Core Team | 4.5.2 |  |
| Software, algorithm | *Brms* |  | 2.23.0 | Bayesian regression interface to Stan |
| Software, algorithm | *Emmeans* |  | 2.0.1 | Estimated marginal means and contrasts |
| Chemical compound | SSR-149415 | Tocris, UK | Cat#: 6195 | Antagonist of AVP V1B receptors |
| Chemical compound | dimethyl sulfoxide (DMSO) | Tocris, UK | Cat#: 3176 | Solvent for SSR-149415 |
| Chemical compound | Conivaptan hydrochloride | Tocris, UK | Cat#: 6830 | Antagonist of AVP V1A / V2 receptors |
| Chemical compound | Methylcellulose | SigmaAldrich | Cat#: M7027 | Solvent for SSR-149415 |
| Antibody | Guinea pig polyclonal anti-AVP antibody | Synaptic Systems | Cat#: 403004 | Primary antibody for immunohistochemistry |
| Antibody | Mouse monoclonal anti-c-Fos antibody | Abcam | Cat#: ab208942 | Primary antibody for immunohistochemistry |
| Antibody | Alexa Fluor® 594 goat anti-guinea [IgG (H+L) antibody](https://rrid.site/data/record/nif-0000-07730-1/AB_2534088/resolver?q=antibody&l=antibody&filter%5b%5d=Catalog%20Number:A11029&i=rrid:ab_2534088-2469544) | Thermo Fisher Scientific | Cat#: A11076 | Secondary antibody for immunohistochemistry |
| Antibody | Alexa Fluor™ 488 [goat anti-mouse IgG (H+L) antibody](https://rrid.site/data/record/nif-0000-07730-1/AB_2534088/resolver?q=antibody&l=antibody&filter%5b%5d=Catalog%20Number:A11029&i=rrid:ab_2534088-2469544) | Thermo Fisher Scientific | Cat#: A11029 | Secondary antibody for immunohistochemistry |
